# Annotations

**Published:** 1898-03-05

**Authors:** 


### Annotations.

### Are We Quite Sure that We are Right.

Dogmatism in medicine has been so fertile a cause of error that professors of the "science of public health," as they are fond of calling it, might perhaps take warning, lest perchance in setting up a sanitary creed to which all must give assent, and enunciating certain rules which all must follow if they would receive the blessing of Whitehall, they also may not be perpetuating fallacies; and in forcing the country to expend enormous sums on sanitary schemes which, admirable as they are, may in a few years be effete, they may not be crystallising error in forms even more solid than dogmas. In a recent speech Viscount Cobham referred to the pressure of the Local Government Board on small places such as Bewdley to carry out expensive sanitation, which, he said, would raise the rates 2s. 6d. in the pound, and he asked that there should not be pedantic insistance on "the very best methods" of water supply or sewage disposal in small places. Finally, he urged that the Local Government Board should set itself to work to find a cheaper system of sewerage for small places than was now insisted on. While we admit the immense amount of good that the country has derived and is deriving from the careful work of this great Government Department, we cannot but agree with some of the strictures which have been made of late years, notably by Dr. Vivian Poore, and from quite another standpoint by Dr. Seaton, in regard to the expenditure involved in carrying out measures imagined to be acceptable to the Board. The matter of the water carriage of sewage is one which is especially urgent. The privy system is of course condemned, and even the pail system, with its much more frequent removal of garbage, has been shown to be related to a larger death-rate from typhoid fever than is found in places where the carriage is by water. But that is by no means a proof that some better system might not be discovered for use in open country places, by which rural authorities might be saved the almost ruinous expenditure required by carrying out extensive schemes for making sewage and then disposing of it. Bricks and mortar are perhaps the most expensive form in which error can be perpetuated.

### Fresh Air for the Cows.

One of the difficulties connected with the growth of modern towns is the supply of all the things that grow in the country, and must be consumed immediately, if they are to be food and not poison. Of these, milk is the most important. The milk supply of towns often leaves a great deal to be desired. Railways bring in milk from considerable distances, and yet it is not enough; and some at least of the milk supply of our cities comes from cows that are kept within their bounds. There is a great deal to be said for the dairyman who keeps his own cows. He can be sure, as the man who gets his milk from a distance cannot be, that typhoid or scarlet fever does not lurk in his milk. He can fulfil the last behests of the sanitary authorities, and be safe from prosecution on any sanitary ground whatever. But, with all his good intentions, he may not be a safe person to deal with. Mr. Warnock, one of the sanitary officials of the city of Glasgow, has recently published a treatise on "Bovine Tuberculosis" which is distinctly uncomfortable reading. Mr. Warnock has, he says, "been engaged during the greater part of his life in the grazing, feeding, rearing, and slaughtering of sheep and cattle, and has had experience of all the cattle diseases and plagues of his time." He declares, in the first place, that tuberculosis, so common now among cattle, is a comparatively new disease, which forty years ago was unknown. Another authority on diseases of cattle is of opinion that at present there are about 800,000 tuberculous cattle in the United Kingdom, or about 5 per cent. of the bovine population. Mr. Warnock thinks that the majority of our cows are constitutionally sound, but holds that a very large proportion of the stall-fed cows of our great cities are tuberculous. "From 20 to 70 per cent. of the stall-fed cows in our towns have been found infected with tuberculosis." This is a dreary look-out for the consumer of city milk. One cannot but spare a pitiful thought for the poor cows themselves, brought in their prime from the fresh air and the green food of the country to the artificial conditions of town life—artificial even for cows; but our pity will for the most part stop at ourselves, destined to unsuspected disease from the tuberculosis which is apt to affect the country-bred cow when it is shut up in a stall in a town.

### The Abortion-Mongers.

The evidence given at a recent trial shows the real inwardness of the numerous advertisements with which too many newspapers defile their columns. It has been often said that advertisements relating to "obstructions" and "irregularities," although very disgusting, and showing how widespread among women is the desire to avoid motherhood, have but little serious relation to the crime of producing or attempting to produce abortion, seeing that the medicines advertised do not usually contain anything which could have "the desired effect." It is clear, however, that the advertisement of a sham abortion drug is but the first step in a long course of crime—the outward bait by which this vile trade is made to flourish. If a woman applies for a medicine because she is "irregular" or suffers from "obstruction," and asks for a drug which is warranted to remove "all female irregularities," and is especially addressed to "married ladies," we may be quite sure that she is seeking a means for procuring abortion. The very terms of the testimonials which purport to have been received by the vendors of these drugs are devised with the object of indicating their object. Why should any woman, after taking a certain drug, say, "In less than twelve hours I was all right, after eighteen weeks of misery and hopelessness"? It is clear that the words "misery and hopelessness" refer to something very different from a mere "irregularity," and that the intention in publishing such statements is to show that she had been pregnant, and that after taking the advertised drug "everything came all right." We admit that the advertisements are carefully worded, and that it would be difficult on the words themselves to show complicity with crime; but the intention is plain enough, and from the evidence recently given we see how the wretched game is played. Women applying for these medicines are advised where to apply in case they fail in their purpose—which, of course, they do—and thus, as the direct result of an advertisement of maybe a useless and innocuous drug, women desiring abortion are introduced to the abortion-monger, whose lair they would otherwise never have been able to find.

